# Enhancement of biocatalyst activity and protection against stressors using a microbial exoskeleton

**DOI:** 10.1038/s41598-019-40113-8

**Published:** 2019-02-28

**Authors:** Jonathan K. Sakkos, Lawrence P. Wackett, Alptekin Aksan

**Affiliations:** 1 Department of Mechanical Engineering, University of Minnesota, Minneapolis, MN 55455 USA; 2 Department of Biochemistry, Molecular Biology and Biophysics, University of Minnesota, Minneapolis, MN 55455 USA; 3 The BioTechnology Institute, University of Minnesota, St. Paul, MN 55108 USA

## Abstract

Whole cell biocatalysts can perform numerous industrially-relevant chemical reactions. While they are less expensive than purified enzymes, whole cells suffer from inherent reaction rate limitations due to transport resistance imposed by the cell membrane. Furthermore, it is desirable to immobilize the biocatalysts to enable ease of separation from the reaction mixture. In this study, we used a layer-by-layer (LbL) self-assembly process to create a microbial exoskeleton which, simultaneously immobilized, protected, and enhanced the reactivity of a whole cell biocatalyst. As a proof of concept, we used *Escherichia coli* expressing homoprotocatechuate 2,3-dioxygenase (HPCD) as a model biocatalyst and coated it with up to ten alternating layers of poly(diallyldimethylammonium chloride) (PDADMAC) and silica. The microbial exoskeleton also protected the biocatalyst against a variety of external stressors including: desiccation, freeze/thaw, exposure to high temperatures, osmotic shock, as well as against enzymatic attack by lysozyme, and predation by protozoa. While we observed increased permeability of the outer membrane after exoskeleton deposition, this had a moderate effect on the reaction rate (up to two-fold enhancement). When the exoskeleton construction was followed by detergent treatment to permeabilize the cytoplasmic membrane, up to 15-fold enhancement in the reaction rate was reached. With the exoskeleton, we increased in the reaction rate constants as much as 21-fold by running the biocatalyst at elevated temperatures ranging from 40 °C to 60 °C, a supraphysiologic temperature range not accessible by unprotected bacteria.

## Introduction

Over the past few decades, key advances in protein engineering have led to exceptional progress in biocatalysis^1–3^, now widely used for industrial scale production of commodity chemicals^4–7^, pharmaceuticals^8–11^, and fine chemicals^12,13^. Generally, enzymes have high catalytic power (k_cat_/k_non_)^14,15^, work under mild reaction conditions (pH, temperature, and pressure) and show substantial selectivity for the substrate, though many enzymes are known to be promiscuous^16^.

Whole cell biocatalysts have several advantages over purified enzymes, including: elimination of the need for protein purification, the ability to catalyze multistep transformations requiring more than on enzyme or pathway, and ease in regenerating cofactors^17^. However, the barrier function of the cell envelope reduces reaction rates, typically 1–2 orders of magnitude lower than that of the purified enzyme^18^. The reduced reaction rates in whole cell biocatalysts can potentially negate the economic gain enabled by the removal of protein purification steps. Improvements in the reactivity, stability, and utility of whole cell biocatalysts can be used to ensure that whole cell biocatalysts are economically competitive with purified enzymes.

There are many strategies for improving the reaction rates in whole cell biocatalysts, ranging from chemical treatments that permeabilize the cell membrane to molecular engineering of the enzyme or membrane^18,19^. Permeabilization of the cell membrane using organic solvents, detergents, salts, freeze/thaw, and electropermeabilization have extensively been used to increase the rate of biocatalysis^20–23^. One caveat to membrane permeabilization treatments is that the specific treatment conditions, such as concentration, time, temperature, pH, etc., are highly empirical and cannot be generalized across organisms nor substrate/product systems. Also, generic destabilization of the membrane may result in loss of cell integrity, limiting longevity and stability. Genetically modifying an enzyme of interest either through rational design or directed evolution can greatly improve reaction rates and enantioselectivity^24–28^. Enzyme overexpression in a bacterial host is also a common method for increasing the concentration of enzyme within the cell and thereby the reaction rate^29^. Lastly, engineering cells for surface display of the desired enzyme negates the need for the either the substrate or product to cross the cell membrane, though may not be suitable for all applications, particularly those requiring metabolic cofactors^30–33^.

Numerous techniques have been studied for stabilizing and improving the function of the whole cell biocatalysts, including flocculation, surface immobilization, and encapsulation^34–37^. A salient feature in all immobilization methods is the ease in separation of the biocatalyst from the reaction mixture, enabling greater utilization of the biocatalyst through repeated use and the potential for a continuous process^38,39^. Flocculation, the addition of a chemical to cause cell aggregation and allow the larger particles to be filtered, does allow for separation of the biocatalyst from the reaction mixture but it cannot be done in a continuous manner. Immobilization to a surface is commonly done using inexpensive carrier materials, such as clay or activated carbon, in combination with a binding agent^40^. This method does not increase the diffusive resistance, since very little, if any coating is applied on top of the cell. Consequently, it confers little protection to the biocatalyst cells as they can detach from the surface, leading to activity loss. Encapsulation, physical confinement of cells within a 3D matrix, in polymeric hydrogels is desirable because it allows for greater protection and isolation from the environment, as well as fine control of the gel structure (e.g. porosity, pore size, and surface functionality)^39,41,42^. These materials can further be functionalized to enhance reactivity^43^, adsorb excess substrate^44^, and even respond to stimuli (pH, temperature, etc.)^45^. One inherent caveat with encapsulation is the increased diffusion length and reduced effective diffusivity, which leads to lower reaction rates^46^.

Using layer-by-layer (LbL) self-assembly, we can deposit nanostructured coatings directly on the cell surface to concurrently immobilize, enhance, and protect the whole cell biocatalyst, while minimizing diffusive limitations. While diverse methodologies have been developed to create LbL assemblies, (electrostatic, hydrogen bonding, covalent, biological, hydrophobic, etc.)^47–51^ the use of oppositely charged polyelectrolyte layers bonded via electrostatic interaction is the most common^52–54^. The layer thickness and porosity can be precisely controlled with the polyelectrolyte charge density, which is a function of pH, ionic strength, solvent quality, temperature, and polyelectrolyte molecular weight^55,56^. LbL coatings can provide protection to whole cells, most notably by improving resistance to elevated temperatures, and by enhancing storage stability when silica was included in the coating^57,58^. Biocatalysis rates up to 5-fold higher (than the uncoated cells) have been reached after LbL coating with polydopamine, reportedly due to improved electron transport, though this is likely not applicable to reactions which do not require redox cofactors^59^. Studies investigating the effect of polycation treatment on the cell membrane of Gram-negative bacteria have shown increased permeability of the outer membrane^60,61^. Since polycations are present in electrostatically-formed LbL coatings, we hypothesized that an LbL coating incorporating a polycation and silica could be used to permeabilize the cell membrane while protecting the whole cell biocatalyst against various environmental stresses, such as high temperature, predation, and acidic pH^62^, leading to enhanced rate of biocatalysis and stability.

This paper presents the construction of a microbial exoskeleton in conjunction with permeabilization of the cell membrane to simultaneously immobilize, protect, and enhance the reactivity of a whole cell biocatalyst while enabling repeated use and providing storage stability. We utilized a hybrid organic/inorganic system for the exoskeleton consisting of the silica precursor tetramethyl orthosilicate (TMOS) and poly(diallyldimethylammonium chloride) (PDADMAC). As a proof of concept for this approach, we deposited the microbial exoskeleton onto *Escherichia coli* cells expressing homoprotocatechuate 2,3-dioxygenase (HPCD), a cytoplasmic enzyme which does not require metabolic cofactors. We showed that with the exoskeleton retaining the enzyme within the cytoplasm while permeabilizing the cell envelope, the reaction rates could be increased by up to 22-fold, approaching levels of the free enzyme. We also showed that at elevated temperatures (40–60 °C), reaction rate constants up to 21-fold higher than that at 25 °C could be reached. This would not be possible without the use of the exoskeleton as at these very high temperatures the membranes dissociate and lose integrity^63^. The microbial exoskeleton also protected the biocatalyst against a variety of external stresses including: desiccation, freeze/thaw, osmotic shock, predation by protozoa, and enzymatic attack.

## Results and Discussion

**Figure 1:**
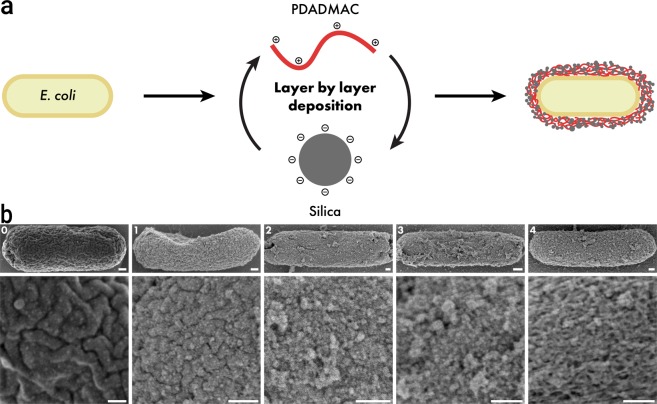
(**a)** Diagram of the layer by layer (LbL) coating process for manufacturing the microbial exoskeleton by alternating the deposition of poly(diallyldimethylammonium chloride) (PDADMAC) and hydrolyzed tetramethyl orthosilicate (TMOS) layers on the cell surface. (**b)** SEM images of *E. coli* with SiO_2_/PDADMAC with 0 (control) up to 4 layers (left to right), illustrating the increasing coverage of the membrane. Bottom panels show the magnified views. Scale bars in all of the panels are 100 nm.

The microbial exoskeleton for *E. coli* was constructed as shown in Fig. 1a. We deposited multiple bilayers on *E. coli* by alternating layers of PDADMAC and silica biosilicification^64–66^. The bacteria were imaged with SEM after deposition of each bilayer confirming gradual coverage of the outer membrane with the application of the initial couple of layers (Fig. 1b). After ≥4 layers, a relatively uniform silica coating was observed, with pores *ca* 10 nm (Fig. S2). SEM images of cells coated with up to 10 layers are presented in Fig. S3. TEM images of an uncoated cell and one coated with 5 layers of PDADMAC/SiO_2_ are shown in Fig. S4. Based on these images, we estimated the thickness of the microbial exoskeleton to be 30–50 nm after 5 layers were added. This was in agreement with the previous work done by Lee *et al*., which measured 5 layers of PDADMAC/SiO_2_ on yeast cells to be 34 nm^67^. Zeta potential measurements showed an alternating change corresponding to the charge of the material deposited (PDADMAC (+), SiO_2_ (−)) in each layer, as expected (Fig. S5). The lack of full charge reversal in the range of layers deposited likely was due to the incomplete surface coverage with <4 layers, which was consistent with the SEM observations. However, after deposition of one bilayer of PDADMAC/SiO_2_, the zeta potential turned positive, which indicated dominance of the PDADMAC charge (+), and insufficient SiO_2_ coverage to screen this charge^68^.

**Figure 2:**
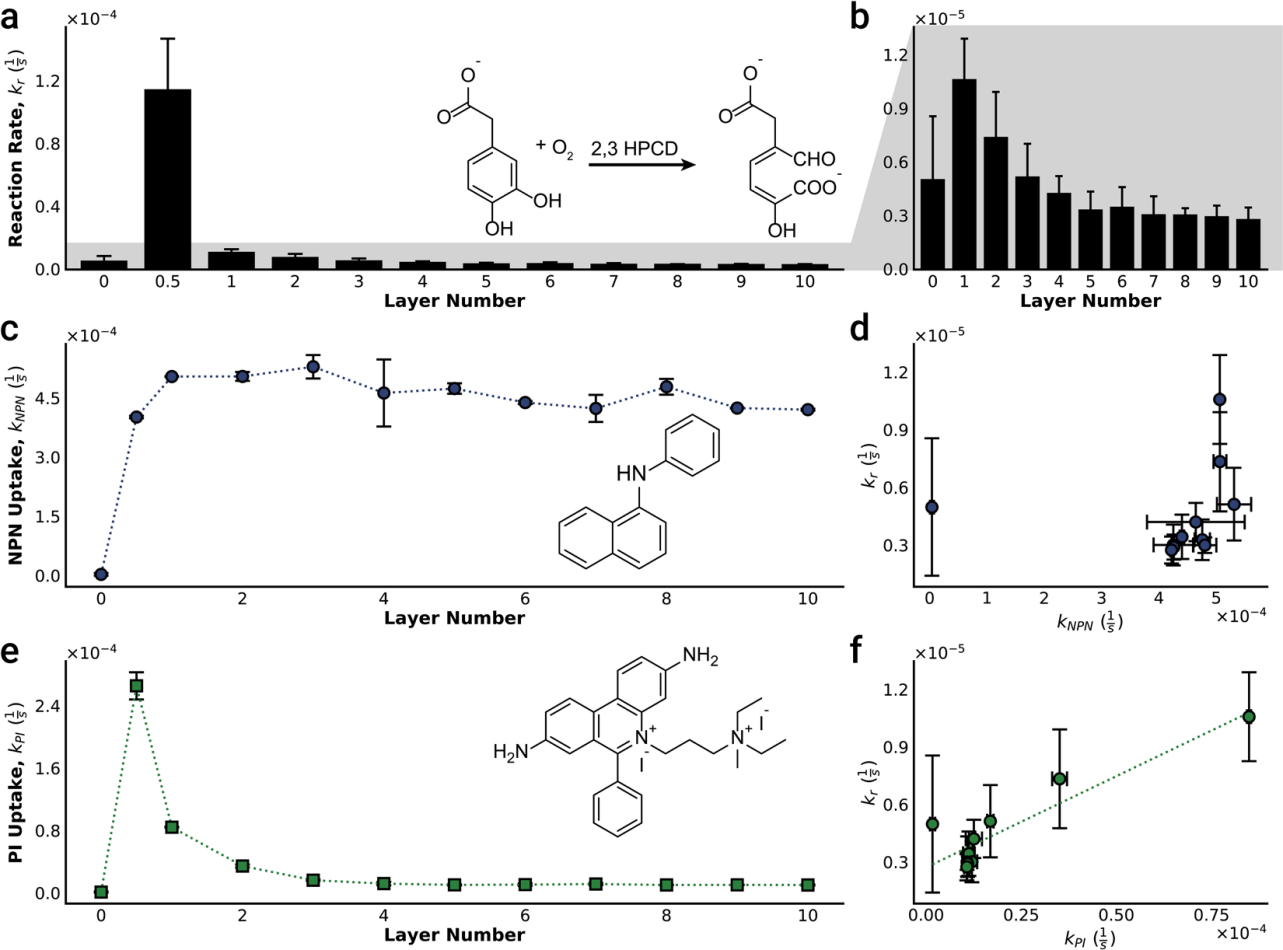
Effect of the number of layers on biocatalysis activity and membrane permeability. (**a**,**b**) Biocatalytic activity: HPCD-catalyzed ring opening of HPAC. (**b**) Layer #0.5 (PDADMAC only) data removed to highlight the small changes observed in rate constant *k*_*r*_ with increasing number of layers. **(c**–**f**) Cell envelope permeability to (**c)** NPN, and (**e**) PI. **(d**,**f**) The change in reaction rate with permeability. Error bars indicate standard deviation, **(a**,**b**) n = 16, (**c**–**f**) n = 4.

To investigate the effect of the microbial exoskeleton on biocatalysis, we followed the ring opening of 3,4-dihydroxyphenylacetate (HPAC) catalyzed by homoprotocatechuate 2,3-dioxygenase (HPCD)^69,70^. This single-step reaction did not depend on other enzymes or metabolic cofactors, was amenable to high-throughput kinetics assays, and thus was a model for the “bag of enzymes” approach to biocatalysis. It should be noted that since the ring opened product of HPAC biocatalysis was measured in the bulk fluid, the reported reaction rate constant (*k*_*r*_) reflected the effects of a combination of the reaction rate within the cytoplasm and the transmembrane transport of the substrate and product. To minimize cell loss during repeated coating and washing steps, in measuring the catalytic activity (described in the materials and methods section in detail) cells were deposited on to 96-well plates and coated with up to 10 layers of PDADMAC/SiO_2_, as shown in Fig. 2a. An increase of 23-fold in *k*_*r*_ was observed when the cells were coated with a single PDADMAC layer (Fig. 2b). *k*_*r*_ was still 2-fold higher after deposition of silica onto PDADMAC, form the first bilayer. In subsequent layers, *k*_*r*_ continued to gradually decline (Fig. 2c), which was attributed to build-up of diffusive resistance. Franz *et al*. report a similar decrease in sulfur/sulfide uptake with increasing layers of coating on *A. vinosum*^48^.

To help explain the enhanced biocatalytic activity results, we quantified the changes in the permeability of the cell envelope using the fluorescent probes 1-N-phenylnaphthylamine (NPN), and propidium iodide (PI)^71^. The cell envelope encompasses three distinct barriers to mass transport; the lipopolysaccharide (LPS) layer, the outer membrane (OM), and the cytoplasmic membrane (CM)^72,73^. While the lipid tails anchoring the LPS insert into the OM, the LPS and OM are functionally dissimilar. The LPS packs tightly via interaction with cations, and predominantly inhibits hydrophobic molecules from entering the cell, and alternatively, the OM primarily limits the diffusion of hydrophilic compounds, which pass through porins^73–76^. NPN is a lipophilic probe which localizes in the lipid bilayer and fluoresces in the presence of a hydrophobic microenvironment but is not typically cell permeable, and thus can be used to assess the permeability of the LPS/OM complex. In contrast, PI must pass through the all three barriers to bind with the nucleic acids in the cytoplasm. Therefore, the difference in the permeabilities of NPN and PI helped distinguish LPS/OM and CM disruption after construction of the exoskeleton^77^. As shown in Fig. 2d, a significant increase in the permeability of the membrane to NPN (k_NPN_) was observed after the initial coating of the cell with PDADMAC (layer #0.5). k_NPN_ did not change appreciably with the addition of more layers, however. These results suggested that PDADMAC disrupted the LPS layer, allowing NPN to access the OM and were consistent with the reports of polycation-mediated membrane disruption^78–83^. We observed no correlation between k_NPN_ and *k*_*r*_ (r^2^ = 0.12), which suggested that the diffusion barrier posed by the LPS/OM was not the rate limiting step for HPAC biocatalysis (Fig. 2e). Permeability of the membrane to PI (k_PI_) also increased rapidly with the addition of PDADMAC but attenuated as a function of increasing number of layers (Fig. 2f). Hong *et al*. proposed that polycations form nanoscale holes within lipid bilayers^84^, which may explain the increase in k_NPN_ and k_PI_. k_PI_ values gradually decreased back to the baseline level after 3–4 layers were deposited, potentially indicating that the PDADMAC/SiO_2_ layers re-stabilized the cell envelope by covering any holes in the CM^72,75,85^. k_PI_ correlated well with *k*_*r*_ (r^2^ = 0.91), indicating that the CM was likely the rate-limiting barrier (Fig. 2g).

**Figure 3:**
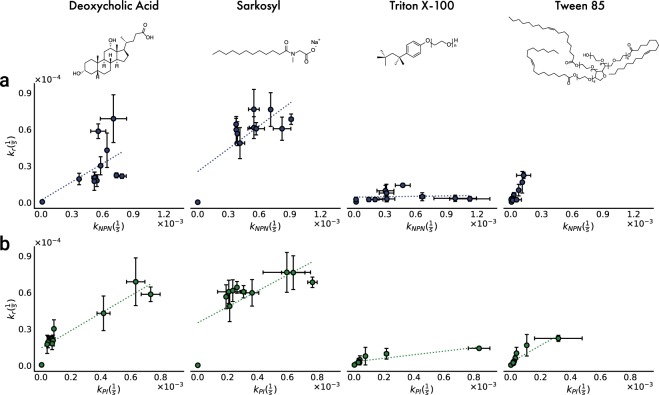
Relationship between the first order reaction rate coefficient for the conversion of HPAC by HPCD, k_r_, and permeability rate constants to NPN (**a**) and PI (**b**), k_NPN_ and k_PI_, respectively, after detergent treatment (1% w/v for 20 minutes), n = 6. Dotted lines indicate linear regression fits to the data. Detergent molecules shown above the respective data. All detergents were used above the known critical micelle concentrations.

**Figure 4:**
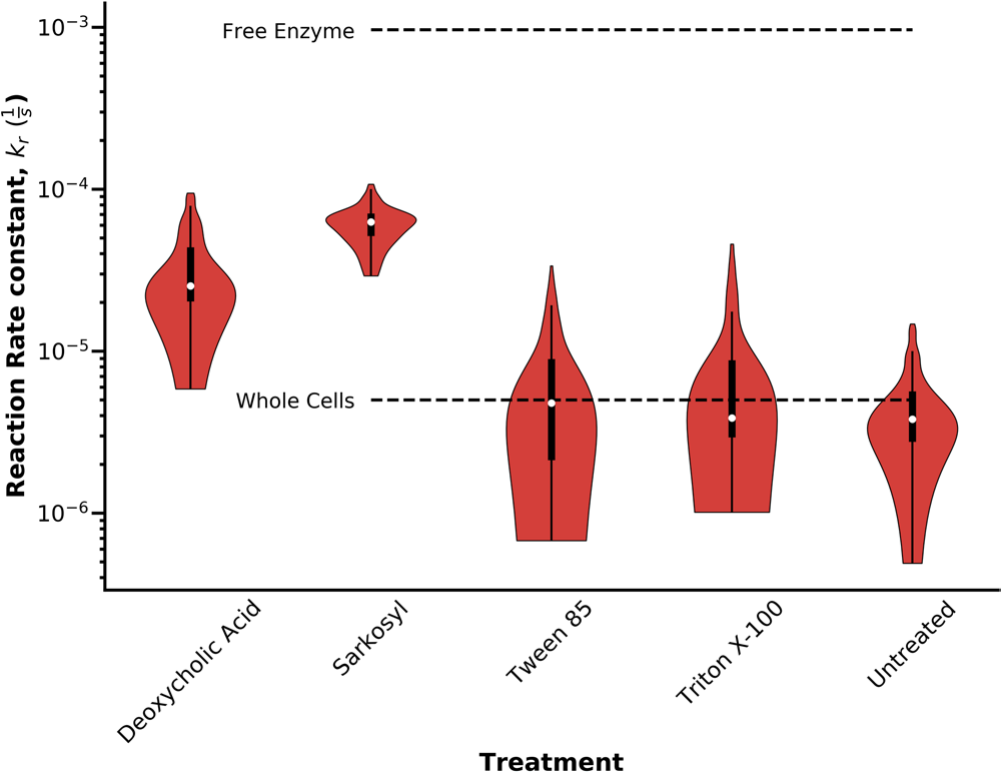
Comparison of the first-order reaction rate constant, *k*_*r*_, of the free enzyme obtained from lysed cells (top dashed line), free whole cells in suspension (bottom dashed line), and LbL coated cells after surfactant treatment (n = 16). The shaded red areas show the sample distribution, the white circles correspond to sample median, and the thick black lines mark the upper and lower quartiles.

We had expected that LbL coating with PDADMAC/silica would both permeabilize and protect the whole cell biocatalyst. While the coating permeabilized the LPS/OM complex (Fig. 2d), the rate of reaction was not significantly enhanced. Based on the cell envelope permeability results, we hypothesized that disrupting the CM would then lead to a significant increase in the rate of biocatalysis, while the exoskeleton ensured mechanical robustness. Several commonly used detergents were investigated for their capability for targeting the CM, including bile salts (Deoxycholic acid, DCA), anionic (Sodium lauroyl sarcosinate, Sarkosyl), and nonpolar surfactants (Triton X-100, and Tween 85) (Table 1). Sarkosyl was chosen because it is known to specifically target the CM^86^, and our results confirmed a large increase (up to 20-fold) in *k*_*r*_ after Sarkosyl treatment independent of the number of layers deposited (Fig. S7a). The permeability measurements showed slight change in k_NPN_ (Fig. 3a), while k_PI_ increased dramatically (Fig. 3b) with respect to untreated samples (Fig. 2f,g), which supported the hypothesis that CM permeability was the main resistance to HPAC biocatalysis. While all the detergents tested led to some increase in k_r_, DCA, and Sarkosyl had stronger effects than Triton X-100, and Tween 85 (Fig. 3). Overall, *k*_*r*_ enhancement inversely correlated with the detergent micelle size with Sarkosyl > DCA > Tween 85 > Triton X-100 (Table 1). Based on the observation of full microbial exoskeleton coverage reached after ~4 layers (Fig. 1b) and the sharp decline in PI uptake between layers 1–4 (Fig. 3b), these results suggested that the larger detergent micelles, particularly Tween 85 and Triton X-100, were excluded from the exoskeleton-coated cells. The size distribution of the exoskeleton pores (Fig. S2) were very similar in size to Triton X-100 micelles (as reported by Robson and Dennis^87^, and Paradies^88^) with hydrodynamic radii of 4 nm, while DCA micelles were much smaller at 1.3 nm^89^. However, when the *k*_*r*_ values of detergent-treated cells were compared with the free enzyme, the relative rates did not surpass 11% of the free enzyme rate, though still improving upon the rates of untreated cells by up to 15-fold (Fig. 4). In order to understand whether this approach was generalizable to other enzymes, we used the microbial exoskeleton on *E. coli* expressing Atrazine chlorohydrolase (AtzA). A 5-fold increase in the rate of hydroxyatrazine production was initially observed (Fig. S6), illustrating the enhanced permeability of the LPS/OM. Detergent treatment did not further enhance the activity, which was likely a result of the increased hydrophobicity of the substrate, atrazine (log k_ow_ = 2.6), vs HPAC (log k_ow_ = 1.1). Coating with 1 layer achieved an activity rate 57.3 ± 4.2% of that with crude extract from AtzA-expressing cells, illustrating that in the absence of the LPS barrier, biocatalysis of a hydrophobic substrate can proceed at rates approaching the free enzyme. Previous studies have shown that molecular mobility in the cytoplasm was an order of magnitude lower than that in water, which suggests that the difference between the permeabilized cells and the free enzyme in this study can be partially explained by hindered diffusion in the cytoplasm^90–92^. In summary, these results showed that Sarkosyl, and to a lesser extent, DCA, were effective in enhancing the rates of HPAC biocatalysis by disrupting the CM, and thus eliminating the primary barrier to diffusion.

**Table 1:** Detergent and micelle properties.

Detergent	Type	MW (Da)	Concentration (mM)	Aggregation Number	Est. Micelle MW (kDa)	Critical Micelle Concentration (M)	Reference
Deoxycholic Acid (DCA)	Bile Salt	393	25.4	22	9	3 × 10^−3^	^112^
Sodium Lauroyl Sarcosinate (Sarkosyl)	Anionic	293	34.1	2	<1		^113^
Triton X-100	Nonionic	1625	6.2	140	228	3 × 10^−4^	^112^
Tween 85	Nonionic	1839	5.4	60 (Tween 80)	110	1.2 × 10^−5^	^112^

**Figure 5:**
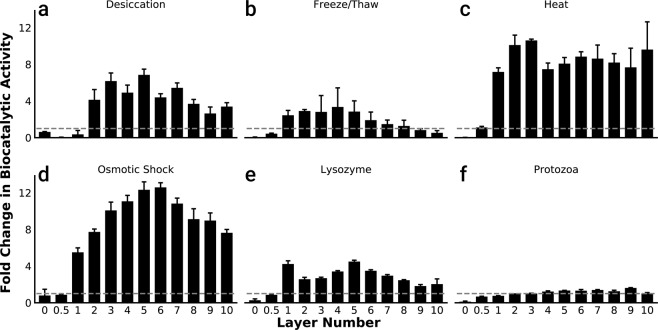
Protection offered by the exoskeleton against a variety of environmental stresses. Exposure to; (**a)** vacuum and desiccation; overnight at 10 Pa vacuum pressure (n = 8), (**b)** freeze/thaw with storage for 1 hr at −20 °C (n = 4), (**c)** high temperature for 30 minutes at 54 °C (n = 4), (**d**) osmotic shock induced with 40% sucrose +1 mM EDTA (1.2 Osm/L) for 20 mins, followed by DI water (n = 4), (**e)** lysozyme at 10 µg/mL +1 mM EDTA for 1 hr (n = 4), (**f)** protozoa for 24 hrs. (n = 8). Data was normalized to untreated controls (grey dashed lines).

The ability of the microbial exoskeleton to protect the biocatalyst against a variety of common stresses was examined (Fig. 5). In order to compare the effect of each stressor separately, the data was normalized to untreated control samples which were originally presented in Fig. 2a. Overall, apart from protozoa exposure, nearly all coated samples had increased *k*_*r*_ after treatment, due to some form of membrane disruption^93,94^. While the specific mechanism of membrane disruption was different in each stress case, the core protective benefit of the microbial exoskeleton was mechanical stabilization of the cell envelope, which led to retention of HPCD and its isolation from the external environment.

Desiccated samples with less than two layers of PDADMAC/silica showed reduced biocatalytic activity while in samples with more than two layers, up to a 7-fold increase in activity was observed (Fig. 5a). Desiccation is known to cause fusion of the lipid membrane, which then becomes leaky after rehydration^95–97^. In the case of this specific stressor, the exoskeleton served to retain HPCD in the cytoplasm after rehydration, opening the route to stabilize and store the biocatalyst in a desiccated state before use. Freeze/thaw treatment resulted in complete loss of biocatalytic activity for samples coated with only PDADMAC, while samples with more layers showed increased activity (while preserving biocatalyst integrity), potentially due to damage to the CM (Fig. 5b). This is expected as unprotected lipid bilayer membranes are known to crystallize, phase separate, and subsequently form non-lamellar lipid structures after freeze/thaw^98^.

Exposure to supraphysiological high temperatures, and osmotic shock yielded the most substantial enhancements in biocatalytic activity (Fig. 5c,d). *E. coli* cells typically alter the composition of their membrane to reduce membrane fluidity to counteract environmental stimuli within physiological temperatures up to 45 °C^94,99^. As expected, heating the uncoated *E. coli* to 54 °C reduced *k*_*r*_ to virtually zero, which is consistent with thermolysis^100–102^. In the meantime, the coated cells benefitted from improved thermal protection conferred by the exoskeleton, and showed an increase in activity in line with increased temperatures, which was consistent with the Arrhenius law^58,103^. Temperatures were selected to illustrate the improved rate of reaction while avoiding denaturation of the enzyme (>60 °C).

Lysozyme/EDTA treatment resulted in near complete loss of activity in uncoated cells (Fig. 5d) as it resulted in cell lysis and leakage of HPCD. EDTA is known to displace divalent metals from the LPS, which allows lysozyme to attack the cell wall^80^. With the cell wall in a weakened state, the cells are susceptible to rupture if the cell envelope is exposed to further stress. The increase in the activity observed in coated cells was likely due to increased membrane permeability as a result of the weakened cell wall coupled with hypotonic treatment, based on the difference in osmolarity between the lysozyme solution (~1 mOsm/L) and phosphate buffer (PB,~100 mOsm/L).

Lastly, the microbial exoskeleton protected the biocatalyst from predation by protozoa over a 24-hour period (Fig. 5f). Samples with at least two layers of the microbial exoskeleton retained more than 90% of their initial activity, while uncoated cells lost nearly all (88% decrease) biocatalytic activity as they were consumed by protozoa. This was consistent with previous work on *E. coli* exposed to protozoa in estuarine water samples^104^.

**Figure 6:**
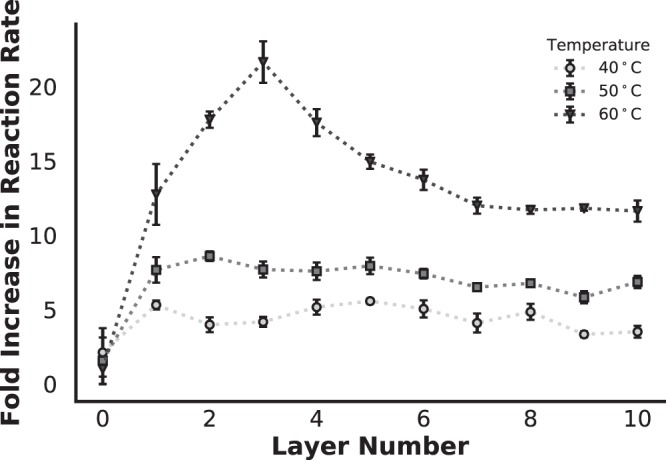
Change in the biocatalysis rate of HPAC by HPCD at elevated temperatures relative to 25 °C (n = 4). The measurements were conducted after 30 mins of exposure.

Due to the costly nature of biocatalysts, it is desirable to increase the reaction rate to decrease the time required to process a given amount of substrate. Based on our results showing protection from high temperatures (Fig. 6), we examined the possibility of using the microbial exoskeleton to facilitate sustained biocatalysis at supraphysiological elevated temperatures. In nearly all cases, a temperature-dependent increase in the reaction rate constant was observed, up to 22-fold, which was consistent with previous work and theoretical predictions (Figs 6, S8)^105^. In the case of untreated cells (0 layers), virtually no increase was observed. This was potentially from loss of cell membrane fluidity due to fusion^93^, and partial thermolysis^100–102^. Note that some uncoated cells were inadvertently lost during repeated assays, which might have amplified the difference. These results proved the feasibility of using the microbial exoskeleton for biocatalysis at supraphysiological temperatures to dramatically increase the throughput and efficiency of a biocatalysis system.

**Figure 7:**
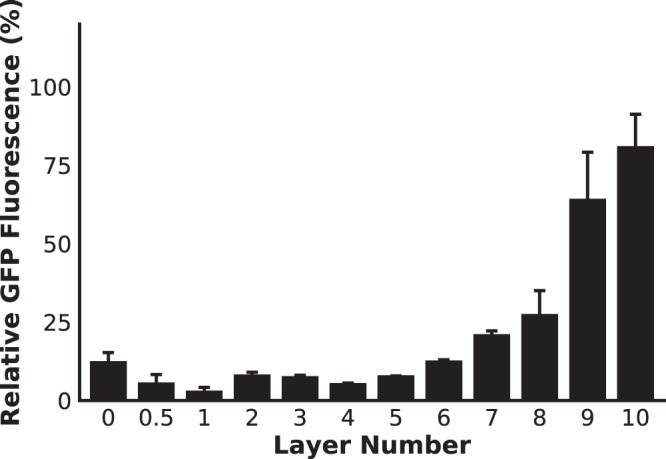
Cytoplasmic protein retention during storage in phosphate buffer (pH 5.8) after 5 days with daily buffer washes to remove leaked protein (n = 16).

In addition to reaching a high the reaction rate, cytoplasmic retention of the biocatalyst over time during repeated use is another critical aspect for industrial efficiency. Polycations (such as PDADMAC) have previously been used as antimicrobial agents because of their ability to permeabilize the cell membrane, allowing for the cell impermeable antibiotics to enter the cytoplasm as well as causing leakage^61,79–81,106,107^. We investigated the retention of GFP after washing and storage to simulate batch reuse cycles of a biocatalyst. After 5 consecutive days of washing and replenishing of the supernatant, we observed a strong dependence on the number of layers (Fig. 7). These results suggested that permeabilizing the cell membrane using PDADMAC caused GFP to escape coated cells more quickly than the uncoated cells, unless a sufficient number of layers (>6) were deposited making up the exoskeleton. Given that the apparent size of the exoskeletal pores was on average 8.5 nm (Fig. S2), we expected some slow leakage of the cytoplasmic proteins, which were confirmed with the observations made between 1 and 5 days (Fig. S9). Based on these results, application of the microbial exoskeleton to other biocatalytic systems will require the consideration of a fundamental trade-off: a greater degree of biocatalyst protection comes at the cost of a reduced reaction rate. The size of the enzyme or complex will have a significant impact on the rate of leakage from the cell and a complex larger than the pore diameter may be retained indefinitely. Thus, the application requirements (e.g. reusability, reaction temperature, and cost) must be taken into account when determining the appropriate number of layers to be used.

LbL deposition of a microbial exoskeleton was found to provide multiple benefits for an entrapped biocatalyst, including immobilization, enhanced reactivity, and protection. Up to a 15-fold improvement in the reaction rate constant was achieved after coating and subsequent detergent treatment. The exoskeleton protected the biocatalyst from a variety of external stimuli and may serve to extend its working life and operating conditions. Lastly, we showed that this system enabled biocatalysis at temperatures up to 60 °C, with improvements in *k*_*r*_ up to 22-fold, which may be of interest for a range of industrial applications.

## Materials and Methods

### Materials

Tetramethoxysilane (TMOS, 98% purity), poly(diallyldimethylammonium chloride) (20 wt % in H_2_O, average M_w_ 200,000–350,000), 3,4-dihydroxyphenylacetate (HPAC), and all other chemicals were purchased from Sigma-Aldrich (Sigma-Aldrich Corp., St. Louis, MO, USA) and used without further purification. Ultrapure water (UPW) was prepared by filtering distilled water using a Milli-Q water purification system (Millipore, Billerica, MA, USA) to a final electrical resistance of >18.2 MΩ/cm.

### Bacterial strains and growth conditions

Homoprotocatechuate 2,3-dioxygenase from *Brevibacterium fuscum* (2,3-HPCD) was expressed in *E. coli* DH5α using a pTRc99A backbone and grown overnight in Luria Broth (LB) with 100 µg/mL ampicillin, as previously described^69,108^. In some cases, GFP-expressing *E. coli* DH5α were grown in LB with 100 µg/mL kanamycin. *E. coli* DH5α (pMD4)^109^ cells expressing Atrazine chlorohydrolase were grown on LB with 30 μg/ml chloramphenicol. *E. coli* cells were harvested by centrifugation at 5,000 × g for 10 mins before resuspension in 50 mM sodium phosphate buffer at pH 5.8 (PB).

### Layer-by-layer deposition

The layer-by-layer deposition was modified from a previously described technique^67^. Cell suspension at either 2 or 20 mg/mL was aliquoted (50 µL) into the wells of 96-well plates. For all wells except those for control, 200 µL of 0.5% PDADMAC in 150 mM NaCl was added and incubated for 5 mins. The plates were centrifuged at 3,000 rpm for 30 mins to deposit the cells into films on the bottom of the plates. The supernatant was removed and the wells were washed 2x with PB before adding 200 µL of 100 mM silicic acid solution for 10 mins, followed by washing 2x with PB. The silicic acid solution was prepared by the hydrolysis of 1 M TMOS in 5 mM HCl for 20 mins before 1:10 (v/v) dilution to 100 mM TMOS in PB at pH 5.8. The process of PDADMAC/SiO_2_ deposition was repeated up to 10 times for the desired number of layers.

### Microstructural analysis

Samples for examining the deposition of material onto the bacteria were made on silicon chip supports. The bacteria suspension was incubated on the silicon for 30 mins before washing off unattached cells with PB. The LbL process was performed by dipping the silicon chips into reservoirs containing either hydrolyzed TMOS, PDADMAC, or PB, as described above, until the desired number of layers were achieved. The microstructure was examined with a Hitachi SU-8230 field emission gun scanning electron microscope (SEM, Hitachi, Ltd., Tokyo, Japan). The samples were fixed for up to 20 hrs with 2% para-formaldehyde (EM grade), 2% glutaraldehyde (EM grade), and 4% sucrose in 0.14 M sodium cacodylate buffer at pH 7.4. After fixation the samples were washed 3x with buffer before adding the postfix solution of 1% OsO_4_ and 1.5% potassium ferrocyanide in 0.15 M sodium cacodylate. The samples were incubated in the postfix solution for 90 mins while occluded from light. After postfixation, the samples were washed 3x and gradually dried in increasing ethanol concentrations of 50%, 75%, 90%, and 100% (twice) for 10 minutes per wash. The samples were then critical-point dried using a Tousimis Model 780 A Critical-Point Dryer before being coated with 5 nm of Ir using a Leica EM ACE600 sputter coater. TEM samples were prepared in the same manner as samples for SEM, but were left in 100% EtOH before embedding in Epon 812 resin overnight and subsequent sectioning. Ultrathin sections (65 nm) were stained with uranyl acetate and lead citrate, and were then imaged on an FEI Tecnai 12 TEM (Field Electron and Ion Company, Hillsboro, OR, USA).

### Surface charge

The ζ potential of coated cells deposited onto plastic coverslips was determined using a SurPASS electrokinetic analyzer (Anton Paar USA, Ashland, VA). Two samples with dimensions 10 × 20 mm were attached to sample holders using double-sided tape. The gap between the samples was adjusted to approximately 120 µm and the streaming current was measured with 1 mM KCl solution at a pressure of 120 mbar.

### Permeability measurement

The permeabilities of 1-N-phenylnaphthylamine (NPN) and propidium iodide (PI) were measured as previously described^61,110^. A 200 µL aliquot of 5 µM NPN and PI was added to each well. The fluorescence intensity of NPN (λ_ex_ = 348 nm, λ_em_ = 408 nm) and PI (λ_ex_ = 535 nm and λ_em_ = 617 nm) were measured for up to one hour with a SpectraMAX Gemini EM plate reader (Molecular Devices, Sunnyvale, CA, USA). The relative changes in fluorescence followed first order reaction kinetics as given by:

1 $$\frac{dF}{dt}={k}_{p}({F}_{max}-F(t))$$

where F(t) is the fluorescence intensity of the probe at time t, F_max_ is the maximum observed fluorescence intensity of the probe, and k_p_ is the first order rate constant. Transforming the fluorescence intensity using the initial fluorescence intensity F(0) and F_max_, we get:

2 $$f(t)=\frac{F(t)-F(0)}{{F}_{max}}$$

And finally, the double normalized fluorescence intensity f(t) is:

3 $$f(t)=1-{e}^{-{k}_{p}t}$$

The experimental data was fit to Equation 3 with a least squares regression to determine the respective first order permeability constants for NPN and PI (k_NPN_ and k_PI_).

### Biocatalytic activity measurement

The activity of HPAC conversion to α-hydroxy-δ-carboxymethyl *cis*-muconic semialdehyde was measured by the absorption of the product at 380 nm (A_380_) as previously described^70^, using a SpectraMAX Plus 384 plate reader (Molecular Devices, Sunnyvale, CA, USA). A 200 µL aliquot of 500 µM HPAC in 50 mM PB at pH 7.5 was added to each well and A_380_ was measured for up to one hour. The plate was shaken in between time points to ensure that the solution was well-mixed and aerated, since oxygen was required for the reaction to occur. Over the course of the experiments, 100 µM or less HPAC and O_2_ were converted, which was well above their respective K_m_ values of 25 and 60 µM, respectively^69,70^. The data was then fit to a first order reaction model of the form:

4 $${C}_{p}(t)={C}_{s,0}(1-{e}^{-{k}_{r}t})$$

C_p_ is the product concentration, C_s,0_ is the initial substrate concentration, and *k*_*r*_ is the first order reaction rate constant.

### Detergent treatment

The coated and uncoated samples were treated with a variety of detergents, including: deoxycholic acid (sodium salt), sodium lauroyl sarcosinate (Sarkosyl), polysorbate 85 (Tween 85), and Triton X-100. A 200 µL aliquot of the desired detergent at 1% concentration (w/v) in water was added to each well and incubated for 20 mins at room temperature before washing 5x with PB.

### Biocatalyst protection

#### Osmotic shock

Samples were exposed to osmotic shock following a previously reported method^111^. PB was added to LbL samples with up to 10 layers before a 50% dilution with a solution containing 1 mM ethylenediaminetetraacetic acid (EDTA) and 40% (w/v) sucrose in PB. The samples were incubated for 10 mins to allow for sucrose uptake. The supernatant was then removed and replaced with ice cold DI water for 10 mins. Finally, the DI water was removed and the samples were washed before activity measurement.

#### Lysozyme treatment

LbL samples were incubated in 1 mM EDTA with 10 µg/mL lysozyme for 1 hr at room temperature, then washed with PB before activity measurement.

#### Desiccation

LbL samples were washed, the supernatant was removed, and the samples were allowed to briefly air dry. The samples were then vacuum dried for 24 hrs at a vacuum pressure of 1 Pa. After drying, the samples were rehydrated with PB before activity measurement.

#### Freeze/Thaw

LbL samples were frozen at −20 °C for 1 hr and washed with PB before activity measurement.

#### Heat treatment

Two distinct heat treatment experiments were conducted. High temperature incubation for 30 mins at 54 °C prior to activity measurement at room temperature. In the second experiment, the samples were assayed at elevated temperatures.

#### Protozoa exposure

LbL samples with up to 10 layers were incubated with mixed protozoa containing *Amoeba, Paramecium, Chilomonas, Stentor, Euglena*, and *Volvox* (Carolina Biological, Burlington, NC) overnight before washing and activity measurement.

#### Protein retention over time

GFP-expressing cells were deposited into a film with up to 10 layers, as described above. The GFP fluorescence of the film was measured with λ_ex_ = 480 nm, λ_em_ = 510 nm, and a 495 nm cutoff filter. The samples were stored at room temperature in PB and washed before measurement.

#### Data analysis and figure generation

All data was analyzed using Spyder 3.3 and Python 3.7. Plots were arranged and diagrams made with Adobe Illustrator CC 2018. The violin plot in Fig. 4 was generated using the Matplotlib 3.0.

## Supplementary information

Supplemental Information

Exoskeleton coated E coli protected from protozoa

E coli scavenged by protozoa
