# Supplementary material for: Enhancement of biocatalyst activity and protection against stressors using a microbial exoskeleton
Source: Sci Rep. 2019 Feb 28;9:3158. doi: 10.1038/s41598-019-40113-8 (PMC6395662; doi:10.1038/s41598-019-40113-8)
Supplement: Supplementary file 1 — Supplemental Information [file 41598_2019_40113_MOESM1_ESM.docx]

Supporting Information

Enhancement of biocatalyst activity and protection against stressors using a microbial exoskeleton

Jonathan K. Sakkos ^a^, Lawrence P. Wackett ^b,c^, Alptekin Aksan ^a,c*^

^a^ Department of Mechanical Engineering, University of Minnesota, Minneapolis, MN 55455, USA

^b^ Department of Biochemistry, Molecular Biology and Biophysics, University of Minnesota, Minneapolis, MN 55455, USA

^c^ The BioTechnology Institute, University of Minnesota, St. Paul, MN 55108, USA

* Corresponding author: (AA) [aaksan@umn.edu](mailto:aaksan@umn.edu)


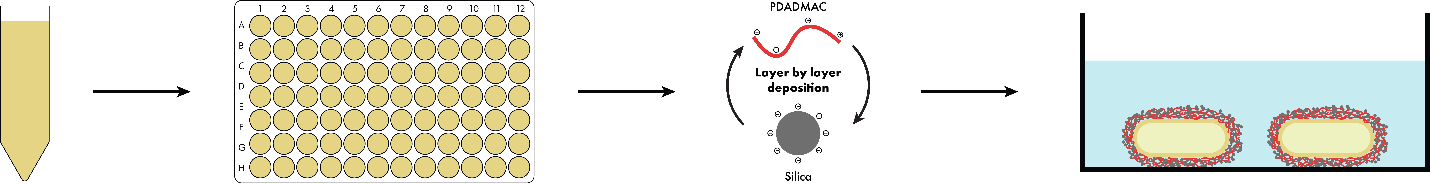


**Figure S1:** Schematic illustrating the coating process, starting with a cell suspension and resulting in coated cells adhered to micro-well plates for analysis.


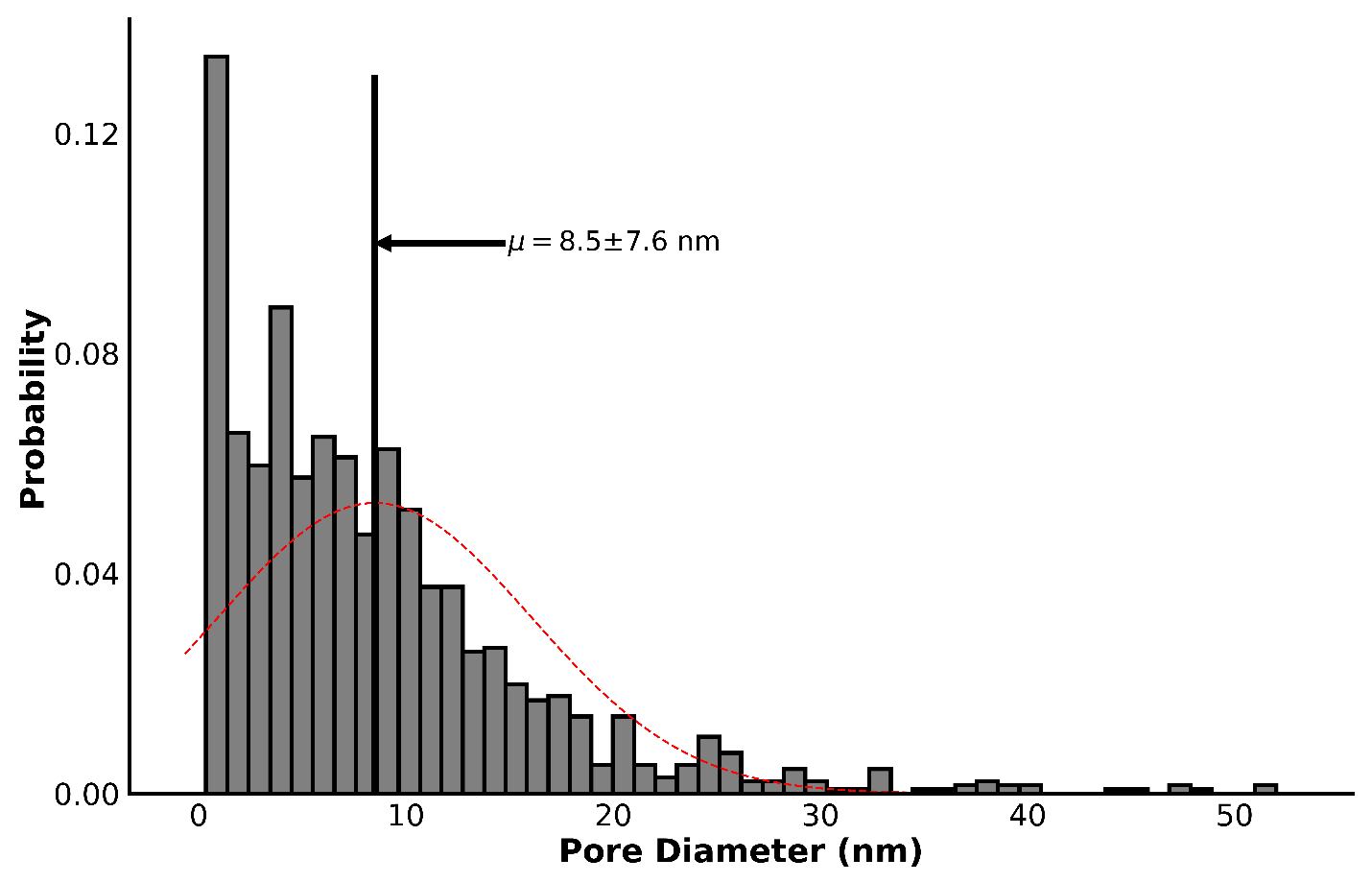


**Figure S2:** Apparent pore diameter measured from SEM images, n > 1,300. Dashed red line indicates a probability density function fit to the mean (8.5 nm) and standard deviation (7.6 nm).


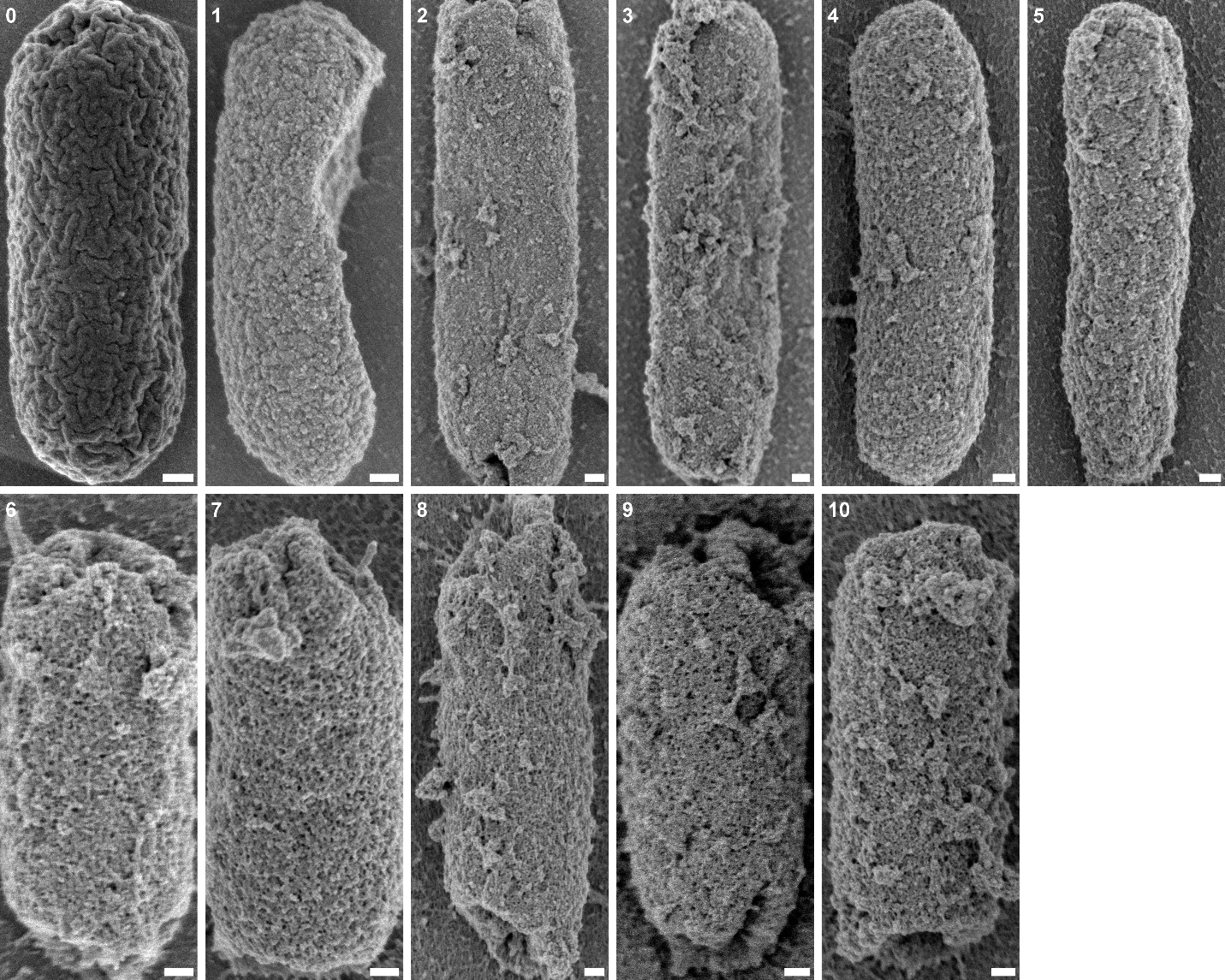


**Figure S3:** SEM images of *E. coli* cells with up to 10 PDADMAC/SiO_2_ layers. Scale bars are 100 nm. Layer number is shown in the upper left corner of each panel.


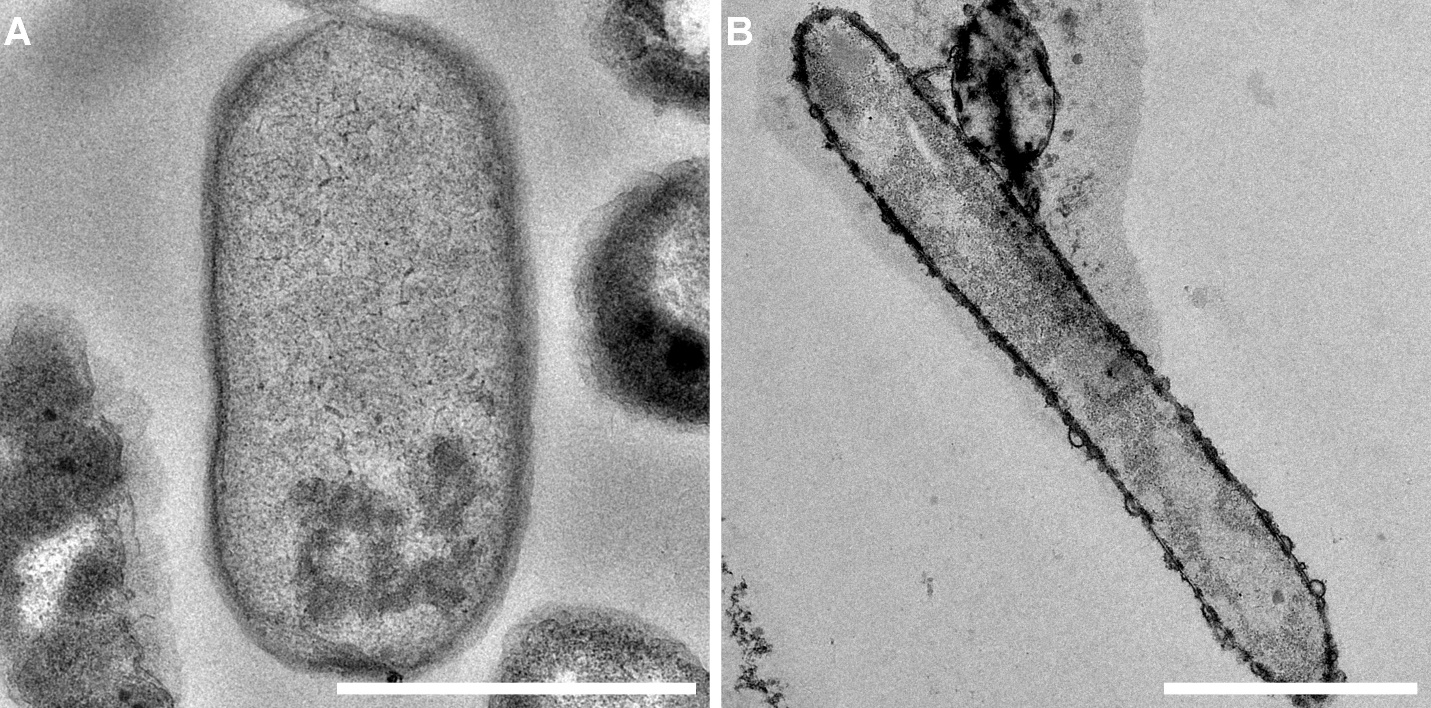


**Figure S4:** TEM image of an uncoated E. coli cell (**A**) and a cell coated with 5 layers of PDADMAC/SiO_2_ (**B**). Scale bars are 500 µm.





**Figure S5:** Zeta potential measurements of coated cells deposited onto plastic coverslips (a) as a function of pH and (b) interpolated at pH 7 (n=4).


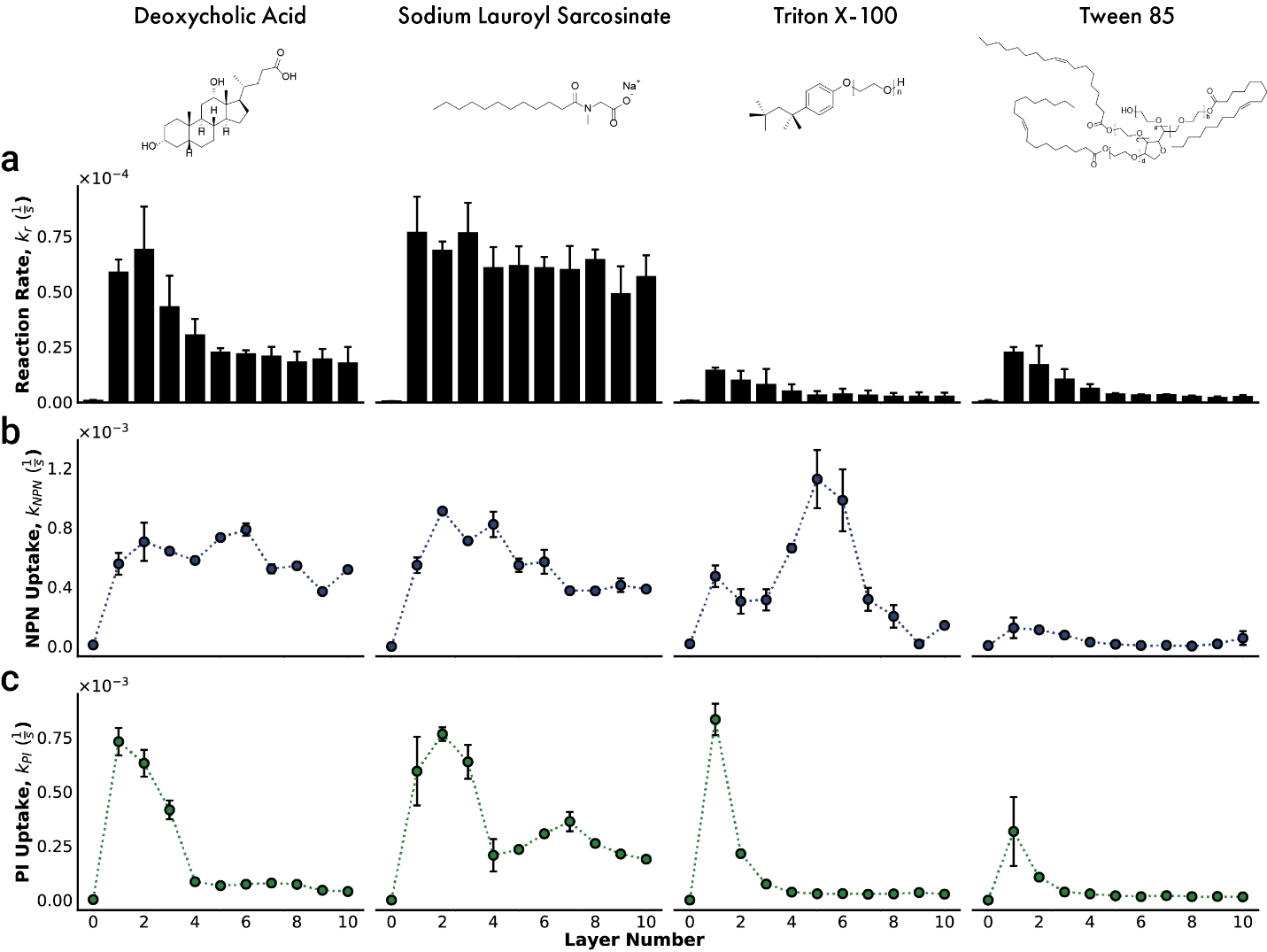


**Figure S6:** Effect of detergent treatment (1% w/v for 20 minutes) on **a)** HPCD biocatalytic activity, **b)** NPN permeability, and **c)** PI permeability. (n=6).


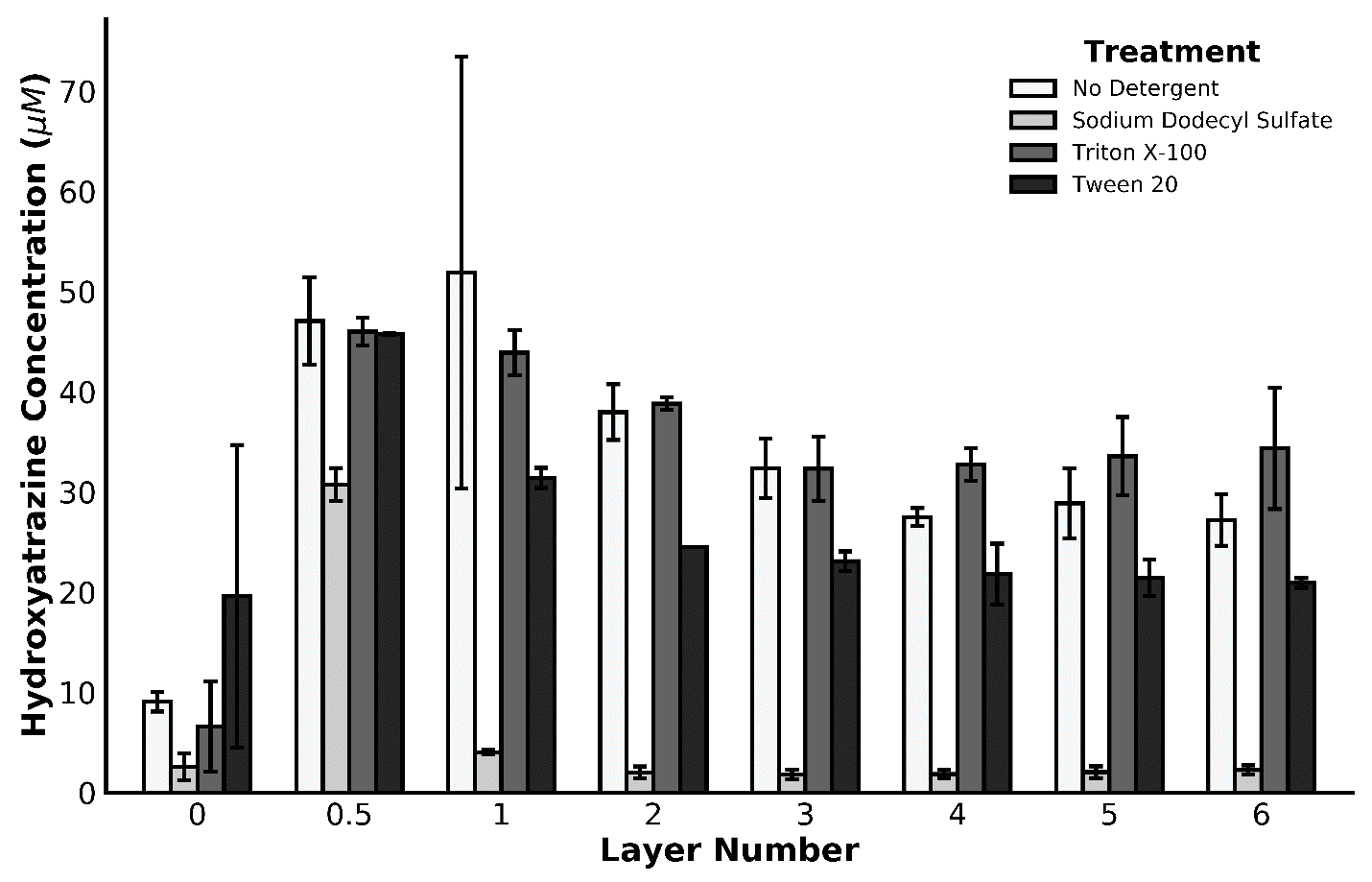


**Figure S6:** Effect of the microbial exoskeleton and detergent treatment on AtzA hydrolysis of 150 µM atrazine into hydrozyatrazine after 1 hour. Detergents treatment was done with 0.5% detergent for 1 hour and subsequent washing before assaying with atrazine.


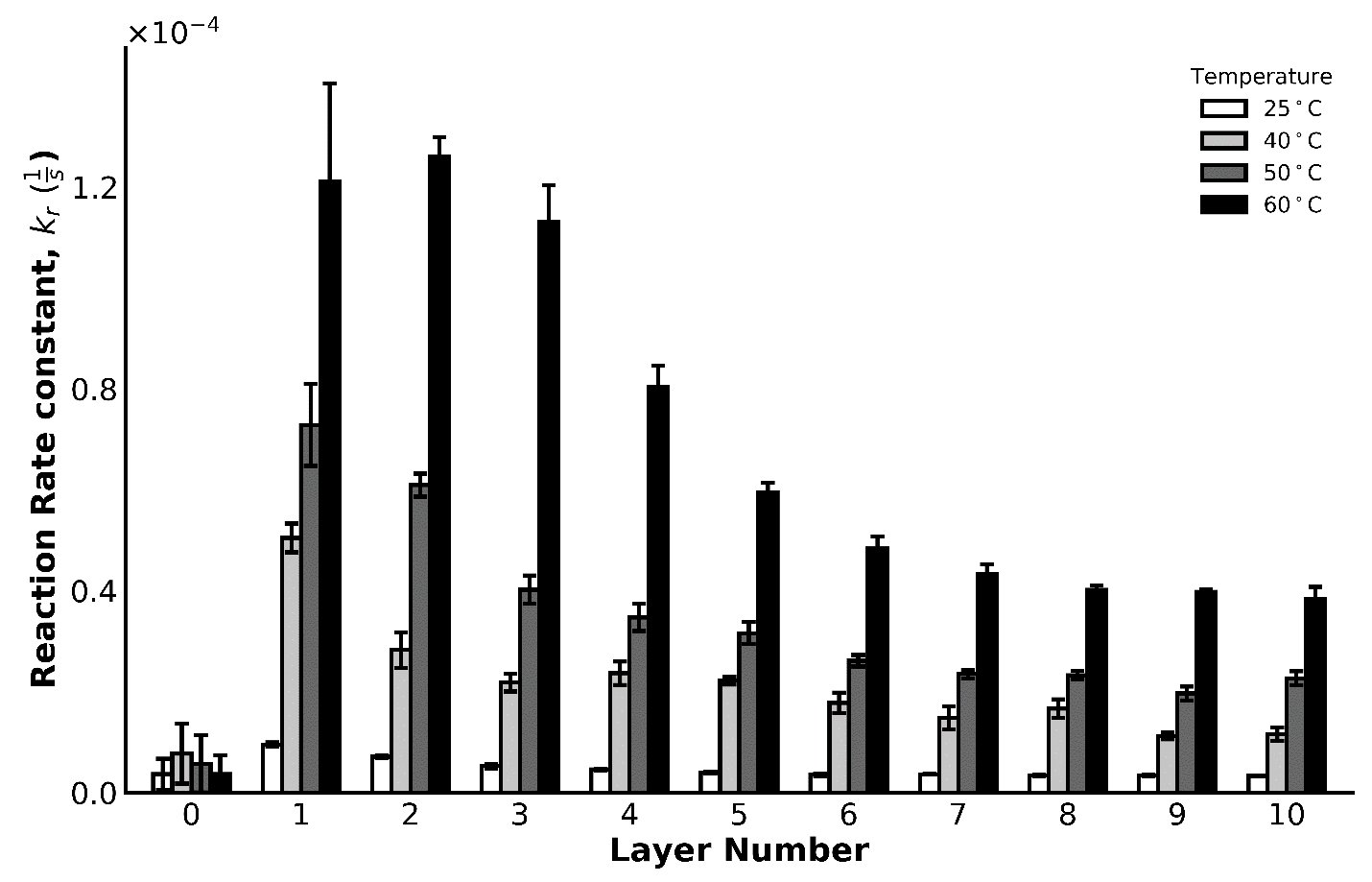


**Figure S8:** Biocatalysis at elevated temperatures over 30 minutes (n=4).


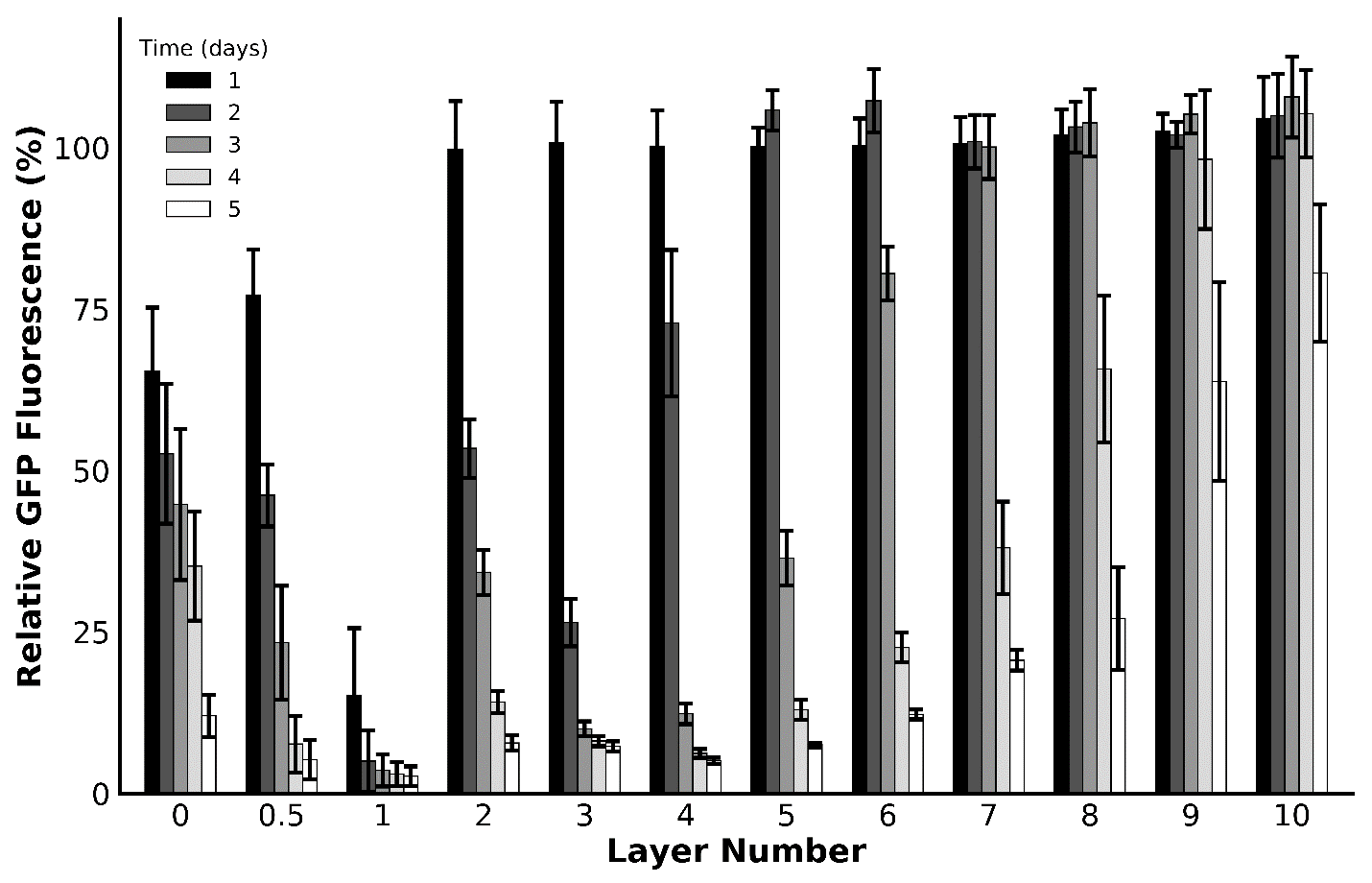


**Figure S9**: Full time course of GFP loss over time, n=16. Data reported in **Figure 6** was day 5 data shown here.


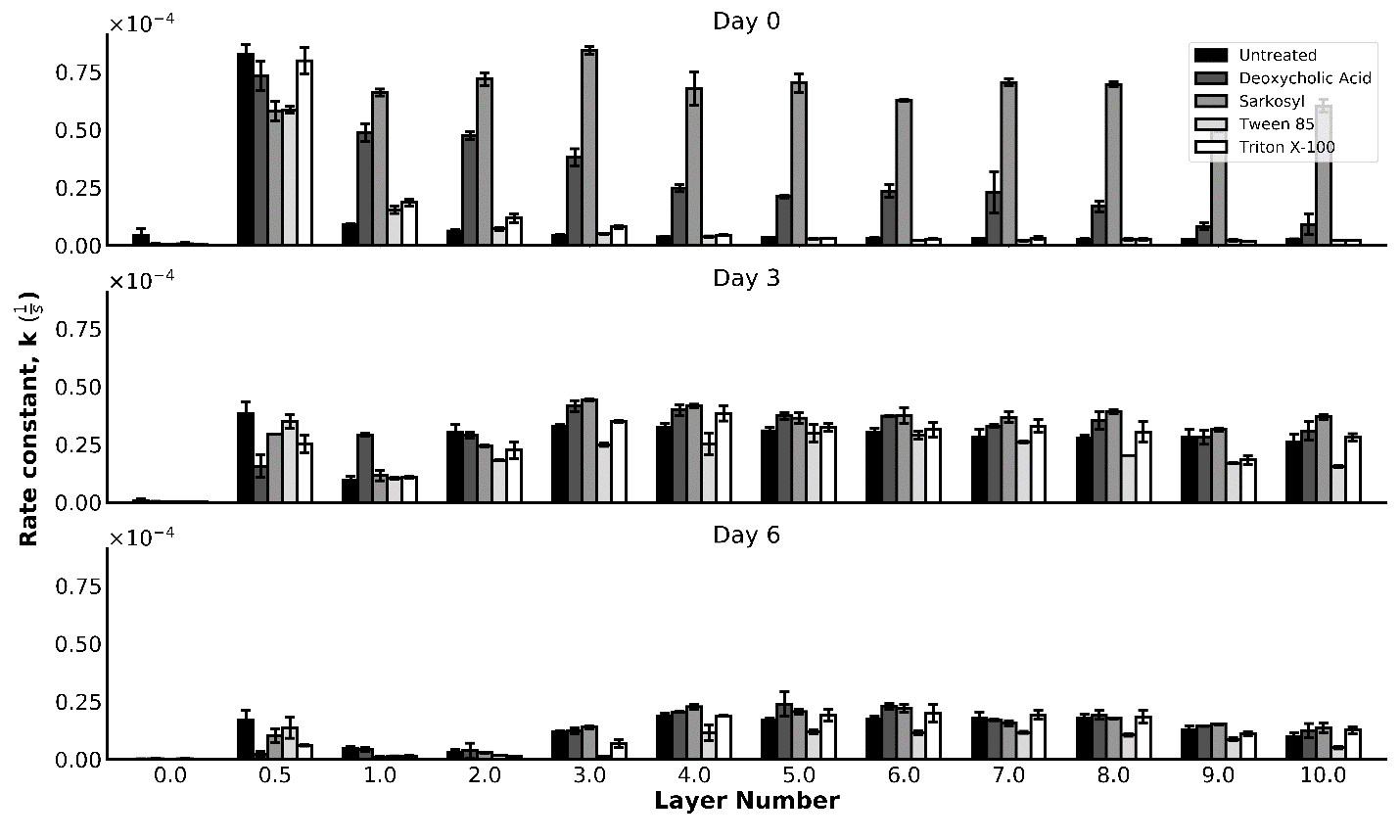


**Figure S10:** Reaction rate constants after detergent treatment and room temperature storage, n=4.
